# Changes of salivary cytokines in head and neck cancer patients following radiotherapy

**DOI:** 10.3389/froh.2026.1969672

**Published:** 2026-10-14

**Authors:** Qingguo Zhao, Juhi Jaiswal, Victor Yu, Ethan Chang, Yu Zhang, Luz Mendoza, Amal A. K. Noureldin, Xiaofang Wang, Fei Liu

**Affiliations:** 1 Cell Biology and Genetics Department, Vashisht College of Medicine, Texas A&M Health Science Center, College Station, TX, United States; 2 Prism Health Dental—East Dallas, Dallas, TX, United States; 3 Department of Public Health Sciences, College of Dentistry, Texas A&M Health Science Center, Dallas, TX, United States; 4 Department of Oral & Craniofacial Biology, College of Dentistry, Texas A&M Health Science Center, Dallas, TX, United States

**Keywords:** dry mouth, head and neck cancer, radiotherapy, salivary cytokines, salivary glands

## Abstract

**Background:**

Radiotherapy for head and neck cancer (HNC) frequently causes irreversible damage to salivary glands, which severely impairs quality of life and is difficult to treat. Preclinical studies indicated that macrophages and related inflammatory processes are essential for the pathogenesis and treatment of this adverse effect. The study on changes of salivary cytokines after radiotherapy in HNC patients is necessary to validate the translational potential of these preclinical findings.

**Methods:**

16 patients with newly diagnosed HNC were enrolled for collecting whole saliva before and one and three months after radiotherapy. Saliva was analyzed using a magnetic luminex assay to quantify 25 cytokines. Changes of salivary cytokine levels and secretion rates between time points were evaluated using paired t-tests.

**Results:**

Chewing-stimulated saliva flow rates significantly decreased one and three months after radiotherapy. Salivary levels of 10 pro-inflammatory cytokines significantly increased at one month and three months, respectively, with 8 of them increased at both timepoints. In terms of the flow rate-adjusted saliva cytokine levels (secretion rates), only CCL2 and GDF15 significantly increased at both time points, whereas 5 other cytokines increased either one month or three months. In mouse salivary glands after radiation, most of these cytokines also significantly increased, which was reversed by a therapy effectively preserved saliva secretion after radiation.

**Conclusion:**

Radiotherapy in HNC patients increased salivary levels and rates of multiple pro-inflammatory cytokines, which encourages further studies of these salivary cytokines as markers for the prognosis and personalized treatment of dry mouth after radiotherapy.

**Clinical relevance:**

The persistent increases of salivary concentrations of many pro-inflammatory cytokines after radiotherapy may exacerbate oral health issues together with the decrease of saliva secretion. The increases in salivary rates of multiple pro-inflammatory cytokines suggest prolonged inflammation and stress in salivary glands, which contributes to long-term dry mouth and are promising therapeutic targets.

## Introduction

Head and neck cancer (HNC) is the seventh most common cancer worldwide, originating predominantly from the mucosal epithelium of the oral cavity, pharynx, and larynx. The majority of HNC cases are head and neck squamous cell carcinomas (HNSCC) (1), with an estimated 940,000 new cases and 480,000 deaths in 2022 globally (2, 3). Radiotherapy is a primary treatment of HNC, either alone or in combination with surgery and systemic therapies. However, radiotherapy frequently damages highly radiosensitive salivary glands, leading to irreversible salivary hypofunction and xerostomia that severely impairs oral health, sleep, speech, swallowing, and overall quality of life (4, 5). Current management options, such as artificial saliva and pharmacologic salivary stimulants, provide only temporary symptomatic relief and do not address the underlying glandular injury and failed regeneration (4, 5).

The lack of effective strategies to prevent or restore salivary gland function after irradiation is mostly due to an incomplete understanding of the underlying cellular and molecular mechanisms. Recently, preclinical studies of radiated skin, lungs, intestine, and salivary glands consistently show radiation-induced alterations in cytokine production and shifts in inflammatory and reparative pathways (6, 7). The cytokine responses to radiation in various healthy tissues are phase-specific: the early phase (hours to days after exposure) responses are characterized by upregulation of pro-inflammatory cytokines and chemokines; during the intermediate phase (days to weeks), cytokines involved in tissue repair, immune modulation, and angiogenesis become prominent; at the chronic phase (weeks to months or longer), persistent cytokine alterations may drive fibrosis, chronic inflammation, and immune dysfunction (8–11). However, whether these preclinical findings are applicable to human patients is largely unclear.

As a noninvasive biofluid, saliva can reflect local mucosal and salivary gland responses in HNC patients undergoing radiotherapy. Cytokines, including chemokines, interleukins, and growth factors, act as master regulators of leukocyte recruitment and activation, cell survival, apoptosis, and tissue remodeling (12). Cytokines in saliva have been explored as biomarkers of oral and systemic oncological or infectious diseases (13). However, data on changes of salivary cytokines after radiotherapy in HNC patiens remain limited. The present study monitored a broad panel of salivary cytokines in a small cohort of newly diagnosed HNC patients before and at one and three months after radiotherapy. We found that radiotherapy in HNC patients decreased the flow rate of chewing-stimulated saliva and increased both salivary levels and rates of multiple pro-inflammatory cytokines. Similar increases of cytokines were found in radiated mouse salivary glands, which were reversed by an effective treatment for preserving saliva secretion. These findings suggest that changes of salivary cytokines contribute to radiation-induced dry mouth and could serve as biomarkers to guide treatment in HNC patients.

## Methods

### Study design and patients

This study is approved by Texas A&M University IRB for saliva collection from patients (IRB2021-1215-CD-FB) and saliva examination (IRB2022-0832M). Patients with newly diagnosed head and neck cancer scheduled to receive radiotherapy were orginally enrolled in a clinical trial of 38% silver diamine fluoride in preventing coronal and root radiation-induced caries (14), and the informed consent was obtained from some of these patients for the collection and examination of saliva including salivary cytokines. Saliva samples were collected before radiotherapy (baseline) and one month and 3 months after radiotherapy. Each patient served as their own control, enabling within-patient comparisons across time points. The inclusion criteria were histologically confirmed HNC, planned curative-intent RT involving salivary glands, and ability to provide saliva samples. Exclusion criteria included active systemic inflammatory or autoimmune conditions and concurrent use of agents known to profoundly alter systemic cytokine profiles. Patients received standard-of-care radiotherapy according to institutional protocols (intensity-modulated radiotherapy with conventional fractionation). Dose, fractionation, and target volumes were planned to encompass primary tumors and involved lymph nodes, with unavoidably high dose exposure to major salivary glands (parotid, submandibular, sublingual).

### Saliva collection and processing

Chewing-stimulated whole saliva were collected using the Saliva-Check BUFFER kit (GC America). To minimize sampling errors, participants were instructed to refrain from eating, drinking (except water), chewing gum, tobacco use, or oral hygiene procedures for at least one hour before sampling (15). Saliva collection was consistently performed as the first procedure after the participant was seated in the examination chair, prior to the clinical examination or any other procedures. Participants were provided with wax gums included in the kit and instructed to chew them for 5 min to stimulate salivary flow. Saliva produced during the first 30 s were discarded. Thereafter, participants expectorated stimulated saliva at approximately 30-second intervals directly into collection cups included in the kit throughout the 5-minute collection period. Then all saliva collected were transferred to 15 mL graduated sterile Falcon tubes to measure volume. Immediately after the measurement, saliva was centrifuged at 3,000 × g for 20 min at 4 °C to remove mucins and debris. The clear supernatants were then transferred to low-binding tubes, aliquoted to minimize freeze–thaw cycles, and stored at −80 °C until analysis.

### Cytokines selected for examination

Based on references and multiplexing compatibilities, we built a custom Human Luminex Discovery Assay (R&D Systems) for examining 25 cytokines with reported roles in radiation damage of health tissues. These cytokines include pro-inflammatory mediators (CCL2, CX3CL1, GDF-15, IFN-*β*, IFN-*γ*, IL-1*α*, IL-1*β*, IL-6, IL-15, IL-17, IL-17C, IL-17E, IL-18, TNF*α*, and TRAIL) (16–18), anti-inflammatory and regulatory factors (IL-10, IL-27, and HGF) (19, 20), cytokines participating in type-2 immunity and fibrosis (IL-4, IL-5, IL-13, and IL-33) (21–23), and cytokines regulating survival, proliferation, and differentiation of myeloid cells (GM-CSF, M-CSF, and IL-34) (24).

### Cytokine measurements

Frozen saliva aliquots were thawed at room temperature just prior to assay. After a brief vortex and, if needed, an additional centrifugation step to remove residual particulates, the supernatant was used for cytokine measurement. Cytokine concentrations were quantified using the Human Magnetic Luminex Assay Kit (R&D Systems, Cat. No. LXSAHM-25), following the manufacturer's instructions. Briefly, standards and samples were incubated with fluorescently encoded, cytokine-specific magnetic beads, followed by detection antibody and streptavidin-phycoerythrin. Plates were read on a Bio-Plex 200 analyzer (Bio-Rad), and analyte concentrations were derived from standard curves generated using a 5-parameter logistic fit.

### Statistical analysis

The distributions of all raw data were examined for both the normality test and the lognormality test using the 4 tests provided by Prism 11. Within-patient changes in salivary cytokine levels between time points (baseline vs. 1 month, baseline vs. 3 months, 1 vs. 3 months) were evaluated using paired t-tests. For each cytokine and comparison, we calculated: The mean paired difference in concentration between time points. The corresponding 95% confidence interval (CI). Interpretation focused on: (i) whether the CI included zero, (ii) the direction and relative magnitude of the mean difference, and (iii) variability and possible influence of outliers (25). Given the small sample size and the large number of analytes and comparisons, no adjustments were made for multiple testing (25). Results are therefore exploratory and descriptive, and P-values should be interpreted with caution.

### Quantitative reverse transcription polymerase chain reaction (qRT-PCR)

RNAs of mouse submandibular glands (SMGs) at 4 or 30 days after radiation were shared from our published studies (7). These RNAs were reversely transcribed with High-Capacity cDNA Reverse Transcription Kit (Applied Biosystems) following manufacturers' protocols. The qPCR was performed with SYBR Green Master Mix (Bio-Rad) on a CFX Opus 96 Real-Time PCR System (Bio-Rad). The primers were synthesized by Invitrogen with sequences retrieved from Primerbank (http://pga.mgh.harvard.edu/primerbank). The qPCR data were analyzed with *Gapdh* as the reference gene using CFX Maestro Software.

## Results

### Radiotherapy led to sustained decreases of saliva secretion in most patients

18 patients were enrolled at the baseline before radiotherapy, but only 16 and 8 patients remained enrolled one month and three months after radiotherapy, respectively. Characteristics of these 16 subjects were summarized in Table 1. The chewing-stimulated salivary flow rates decreased in 12 out 16 patients by one month and in 7 out 8 patients by three months, and the decreases across all patients were significant at both post-radiotherapy timepoints without significant difference between them (Figure 1).

**Table 1:** Characteristics of subjects.

Characteristics	Categories	Numbers
Age (years)	41–59	8
	60 and above	8
Sex	Male	14
	Female	2
Cancer locations	Oral cavity	5
	Oropharynx	6
	Hypopharyngeal	1
	Data not available	4
Tumor resection	Yes	6
	No	6
	Data not available	4

**Figure 1:**
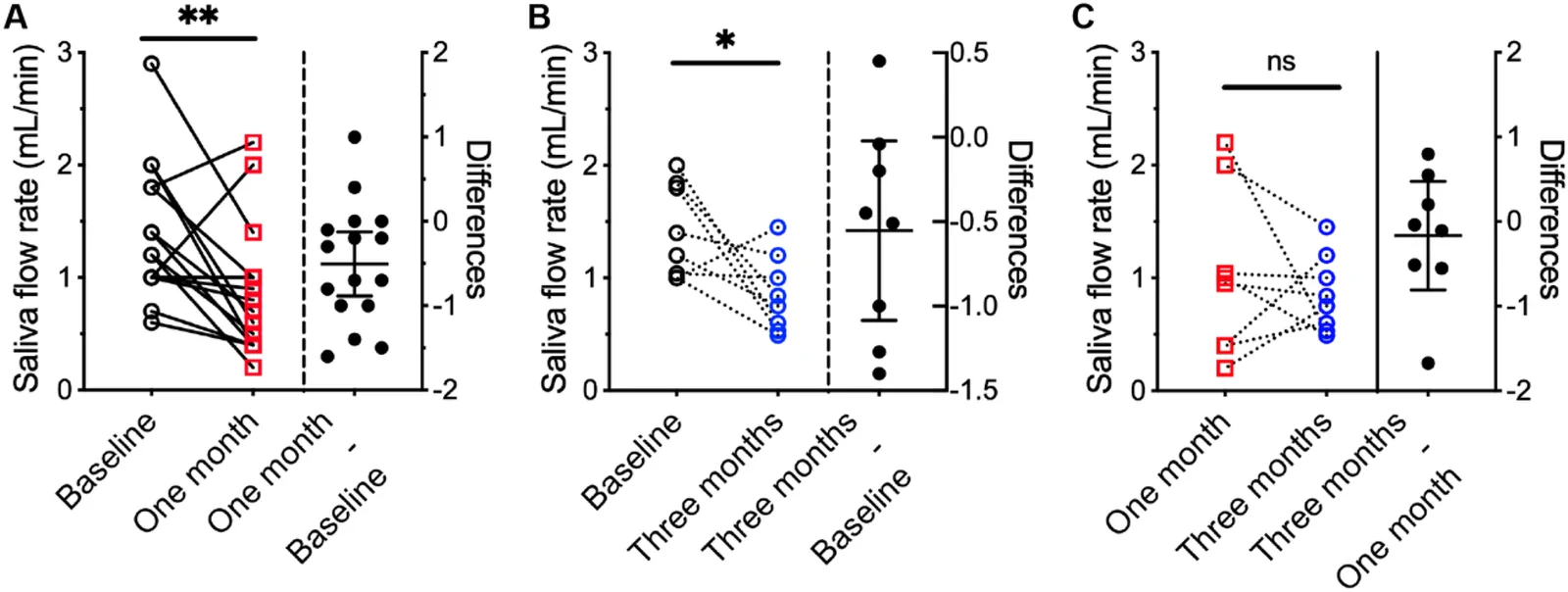
**(A–C)** Chewing-stimulated salivary flow rates (mL/min) were measured at baseline and one or three months after radiotherapy in patients with head and neck cancer. Since distributions of all data passed the lognormality test but the one-month data did not pass the normality test, ratio paired t-tests were performed to compare baseline vs. one month after radiotherapy **(A)**, baseline vs. three months after radiotherapy **(B)**, and one month vs. three months after radiotherapy **(C)**. *N* = 16 in A and *N* = 8 in **B-C**. Differences are shown as individual values and Mean ± SD. **: *p* < 0.01, *: *p* < 0.05, ns: not significant.

### Radiotherapy persistently increased saliva levels of multiple pro-inflammatory cytokines

The concentrations of all cytokines at all three time points varied greatly between patients, and their distributions passed the lognormality test but not the normality test. One month after radiotherapy, among 25 cytokines examined, saliva levels of 10 cytokines significantly increased, including chemokines (CCL2 and CXCL1), growth factors (M-CSF, HGF, and GDF15), and pro-inflammatory factors (IL1*α*, IL1*β*, IL4, IL6, and TNF*α*) (Figure 2). Three months after radiotherapy, except for M-CSF and IL1*β*, saliva levels of 8 of these 10 cytokines remained higher than the baseline without significant differences compared with those by one month. Meanwhile, two additional pro-inflammatory cytokines, GM-CSF and IL18, became significantly higher in saliva than the baseline. Salivary levels of 13 other cytokines examined did not significantly change at either timepoints (Supplementary Figure S1).

**Figure 2:**
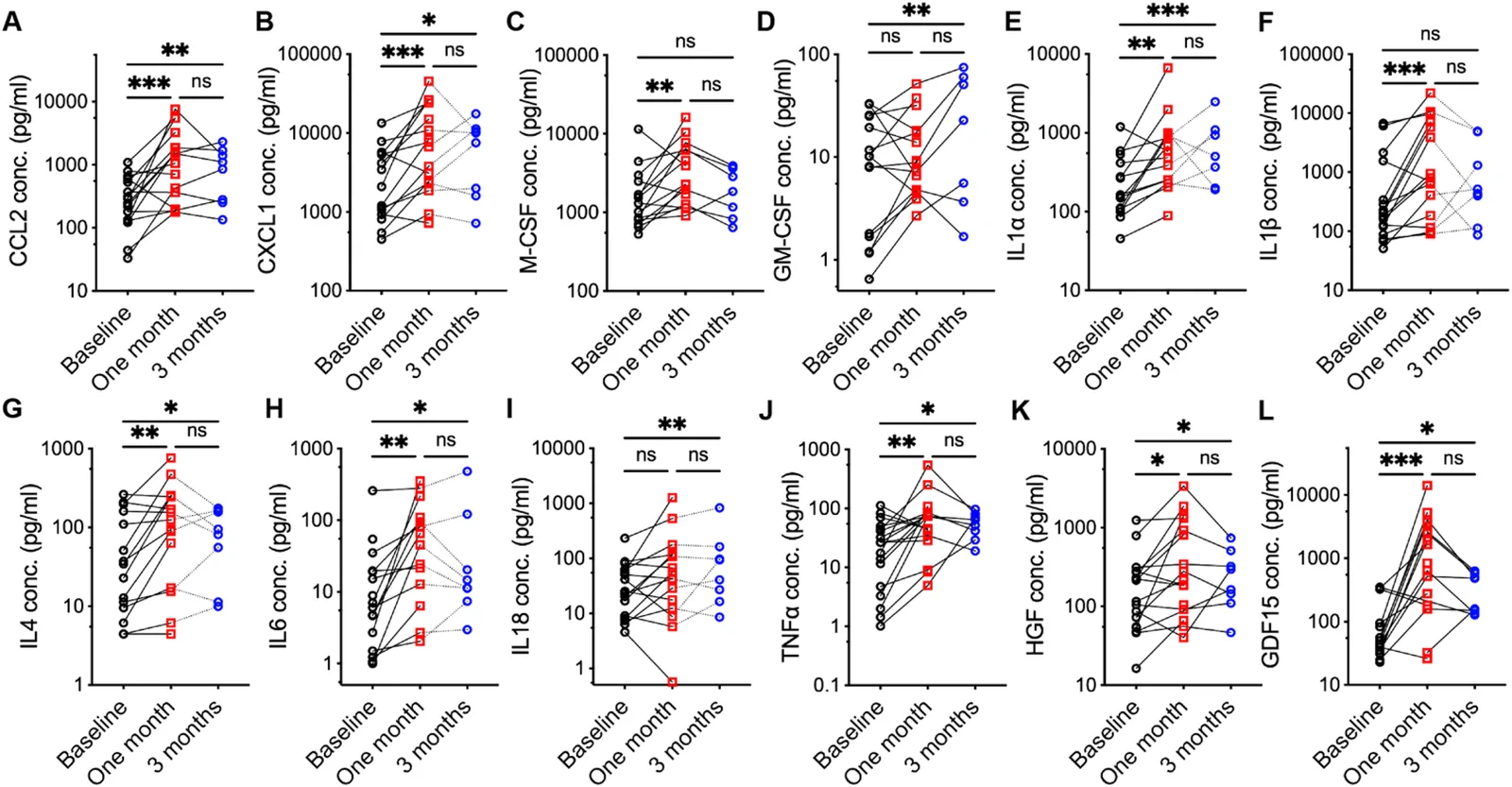
**(A–L)** Salivary levels of cytokines significantly changed after radiotherapy. Cytokine concentrations (conc.) were quantified using the Human Magnetic Luminex Assay kit in saliva from 16 patients at baseline and by one month after radiotherapy and 8 patients by three months after radiotherapy. Since distributions of all data passed the lognormality test but not the normality test, geometric means were compared between each time points using a one way mixed-effects model. ***: *p* < 0.001, **: *p* < 0.01, *: *p* < 0.05, ns: not significant.

### Radiotherapy increased flow rate-adjusted salivary levels of fewer pro-inflammatory cytokines

Since the saliva flow rate decreased significantly after radiotherapy, the flow rate-adjusted salivary cytokine levels (pg/min, abbreviated as salivary rate) are better indexes to reflect cytokine expression and immune activity in salivary glands (26, 27). Among 25 cytokines examined, only 7 pro-inflammatory cytokines showed significant increases in salivary rates either one month or three months after radiotherapy, and two of them (CCL2 and GDF15) increased at both timepoints (Figure 3). The salivary rates of IL1*β* and IL6 significantly increased only by one month after radiotherapy, and those of GM-CSF, IL1*α*, and IL18 elevated only by three months. Salivary rate of 18 other cytokines examined did not significantly change at either timepoints (Supplementary Figure S2).

**Figure 3:**
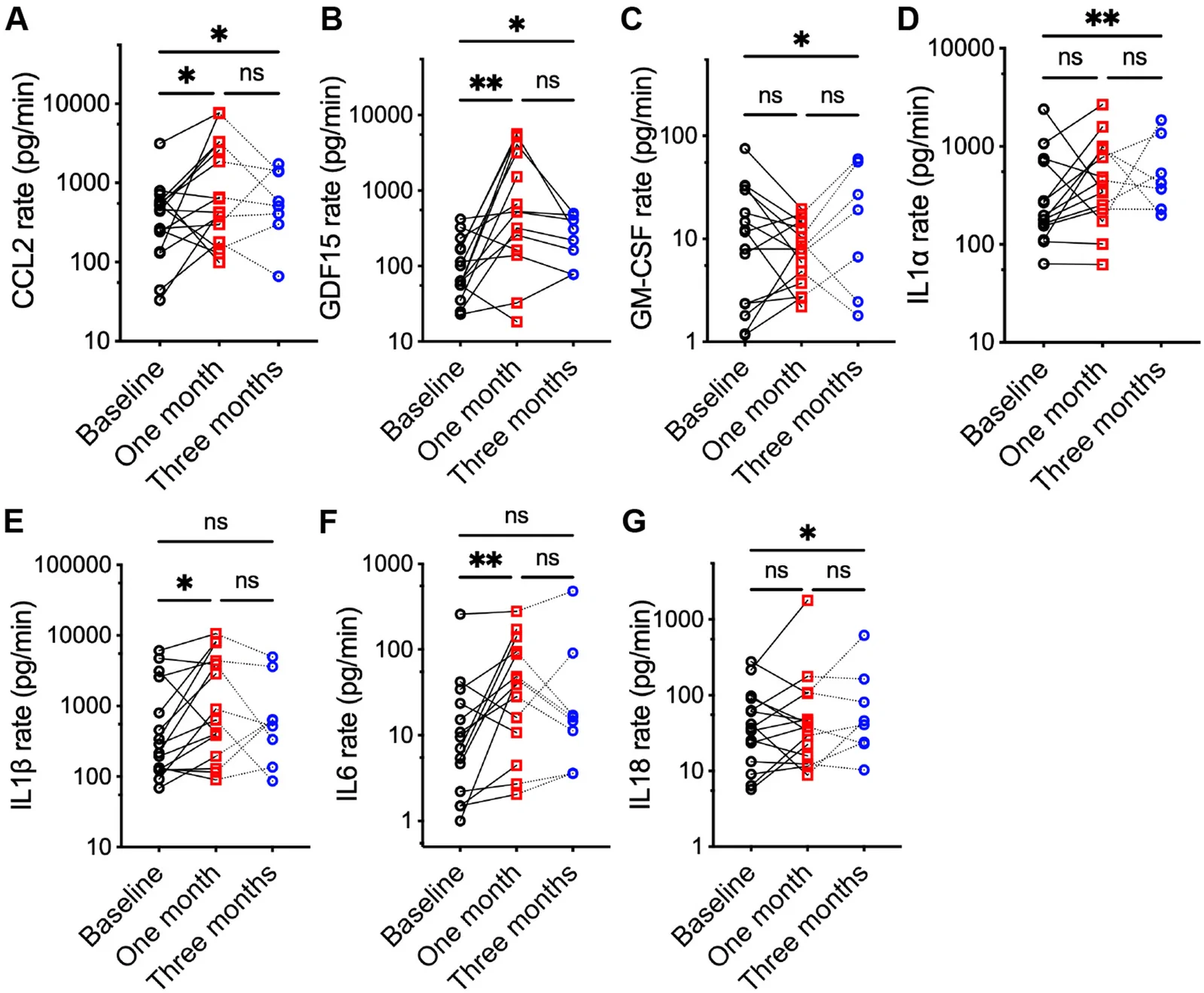
**(A–G)** Rate-adjusted salivary levels of cytokines significantly changed after radiotherapy. Rate-adjusted salivary levels of cytokines were calculated by multiplying salivary cytokine concentrations with saliva flow rate. Since distributions of all data passed the lognormality test but not the normality test, geometric means were compared between each time points using a one way mixed-effects model. ***: *p* < 0.001, **: *p* < 0.01, *: *p* < 0.05, ns: not significant.

### Related changes of cytokine genes in mouse salivary glands after radiation and regenerative therapy

Our group has reported that Gdf15 and other cellular senescence markers were remarkably upregulated in mouse salivary glands by 7 days after radiation; conversely, the preservation of mouse salivary gland function after radiation by Hedgehog agonists repressed the acute elevation of GDF15 (28), which is largely mediated by maintaining anti-inflammatory and pro-regenerative tissue resident macrophages (29). More recently, our RNA-sequencing data indicated that multiple pro-inflammatory cytokines were remarkably upregulated in mouse submandibular glands (SMGs) by 30 days after radiation; conversely, extracellular vesicles from iPS cell derived mesenchymal stem cells (iMSC-EVs) efficiently preserved mouse salivary glands function after radiation and repressed the chronic elevation of these pro-inflammatory cytokines (7). To evaluate the biological relevance of cytokines with significantly changed rate-adjusted salivary levels after radiotherapy in HNC patients, we analyzed mRNA levels of these cytokines in previously collected SMG RNA samples from C57BL/6 mice 4 and 30 days after radiation with or without weekly intra-SMG iMSC-EV treatment from day 1 to 14 (Figure 4A). The qRT-PCR analysis indicated that at both time points, the mRNA levels of Ccl2, Gdf15, IL1a, IL1b, and IL6 were significantly increased by IR, whereas the iMSC-EV treatment inhibited such increases by day 30 except for IL1a (Figures 4B–F). On the other hand, mRNA levels of GM-Csf and IL18 were not significantly affected by IR or iMSC-EV treatment (Figures 4G,H). These data suggested that most changes of these cytokines in human saliva are likely related to their expression in radiated salivary glands, and the treatments effectively preserve saliva secretion after radiation therapy reversed the increases of these cytokines in radiated salivary glands.

**Figure 4:**
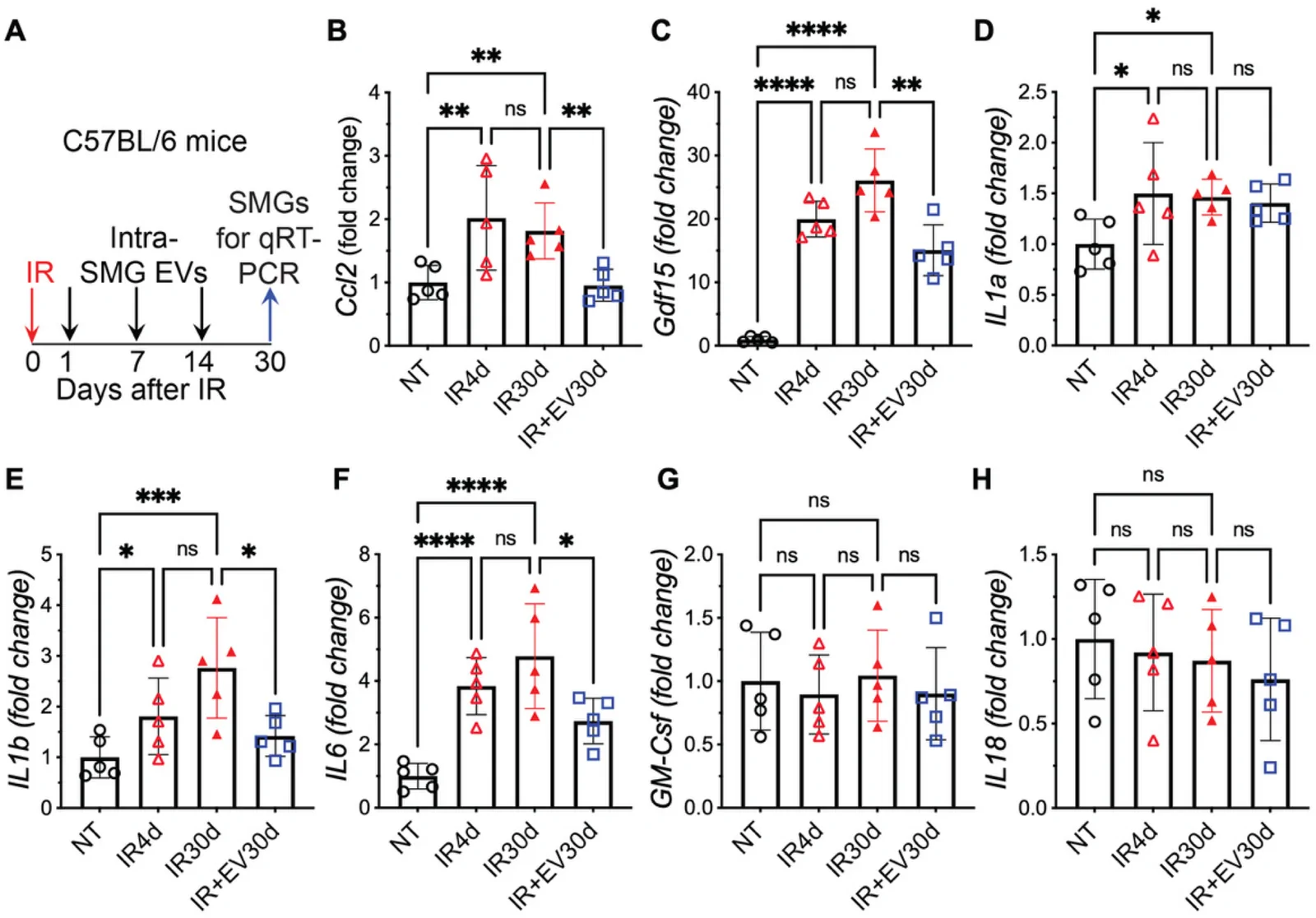
mRNA levels of cytokines in mouse SMGs after radiotherapy and iMSC-EV treatment. **(A)** Experiment design. **(B–H)** qRT-PCR analyses of cytokines in mouse SMGs after radiotherapy and iMSC-EV treatment. *N* = 5. ****: *p* < 0.0001, ***: *p* < 0.001, **: *p* < 0.01, *: *p* < 0.05, ns: not significant.

## Discussion

In this study, we found that salivary concentrations of 8 cytokines (CCL2, CX3CL1, GDF15, HGF, IL-1*α*, IL-4, IL-6, and TNF*α*), increased both one month and three months after radiotherapy, whereas 4 other cytokines increased only by one month (M-CSF and IL-1β) and by three months (GM-CSF and IL-18), respectively. Among these 12 cytokines, CCL2 and CX3CL1 recruit leucocytes; IL-1*α*, -1β, -4, -6, -18 and TNF*α* promote apoptosis, cellular senescence, and inflammatory; M-CSF and GM-CSF regulate differentiation and activation of myeloid cells; GDF15 participates in senescence of epithelial and endothelial cells after radiation (30, 31); whereas HGF inhibits apoptosis and cellular senescence in radiated tissues (32, 33). Conversely, the salivary concentrations of anti-inflammatory IL-10 and IL-27 did not significantly change after radiotherapy. After the adjustment with saliva flow rate, cytokine levels (secretion rates) of CCL2 and GDF15 were still significantly increased at both time points, those of IL1*β* and IL6 significantly increased only by one month, and those of GM-CSF, IL-1*α*, and IL-18 elevated only by three months, whereas those of CX3CL1, M-CSF, IL-1*β*, IL-4, and HGF did not significantly change. These data indicate that the overall change of saliva cytokine profile after radiation is dominated by the increases of factors promoting inflammation and cellular senescence, which might contribute to the radiation-induced dental caries and tooth decay (34, 35).

As indicated by a meta-analysis including 40 studies and encompassing 1,280 patients with oral cancers and 1,254 healthy controls, significantly higher levels of salivary IL-1β, IL-6, IL-8, IL-10 and TNF-α were observed in patients with oral cancers (36). These cytokines, especially TNF-α, are associated with cancer progression and metastasis and could be partially derived from cancer microenvironment. However, in recurrence-free cancer survivors including those with oral cancers, radiation therapy but not surgery alone significantly increased salivary levels of many cytokines, including those mentioned above (37). Similar increases after radiation therapy have been reported by multiple other studies (38, 39) and confirmed by our data above. These findings indicated that despite of higher baseline levels of many cytokines in saliva from head and neck cancer patients than healthy controls, radiation therapy significantly and persistently increased them even after normalization with the decreased saliva secretion.

In animal models, we and other labs found that the damage of tissue resident macrophages and persistent inflammation contribute to radiation-induced salivary gland dysfunction and are promising therapeutic targets (29, 40, 41). In SMGs from wild type C57BL/6 mice after local radiation, similar increases of 5 pro-inflammatory cytokines (CCL2, GDF15, IL1a, IL1b, and IL6) were found at both day 4 and day 30, which was inhibited by effective treatment to preserve salivary gland function. These findings together suggest that in HNC patients treated with radiation therapy, salivary cytokines are potential non-invasive biomarkers for predicting dry mouth, stratifying pateints for macrophage-targeting therapies, and monitoring efficacy of such therapies.

It should be noted that our study only enrolled a small number of patients due to the limited funding. Therefore, above analyses are exploratory and mostly descriptive, and the findings should be interpreted cautiously and validated in larger cohorts.

## Conclusions

Radiation therapy in head and neck cancer patients leads to sustained decrease of chewing-stimulated saliva flow rates and increases of multiple pro-inflammatory cytokines in saliva. Similar changes of many of these cytokines were found in mouse salivary glands after radiation, which was reversed by therapies effectively preserved saliva secrtion. The present exploratory study provides a proof-of-concept that the salivary cytokine profiling may inform more precise and personalized interventions to mitigate radiation-caused dry mouth in HNC survivors.

## Data Availability

The original contributions presented in the study are included in the article/Supplementary Material, further inquiries can be directed to the corresponding author.

## References

[1] Johnson DE. Head and neck squamous cell carcinoma. Nat Rev Dis Primers. (2020) 6:92. 10.1038/s41572-020-00224-3 33243986 PMC7944998

[2] Liu Y Zhang N Wen Y Wen J. Head and neck cancer: pathogenesis and targeted therapy. MedComm (2020). (2024) 5:e702. 10.1002/mco2.702 39170944 PMC11338281

[3] Smith CDL. Head and neck cancer incidence is rising but the sociodemographic profile is unchanging: a population epidemiological study (2001–2020). BJC Rep. (2024) 2:71. 10.1038/s44276-024-00089-z 39301277 PMC11408244

[4] Jasmer KJ Gilman KE Munoz Forti K Weisman GA Limesand KH. Radiation-induced salivary gland dysfunction: mechanisms, therapeutics and future directions. J Clin Med. (2020) 9:4095. 10.3390/jcm9124095 33353023 PMC7767137

[5] Blitzer GC Paz C McCoy SS Kimple RJ. Radiation-therapy related salivary dysfunction. Semin Radiat Oncol. (2025) 35:278–84. 10.1016/j.semradonc.2025.02.006 40090753 PMC11911547

[6] Wu Y. Contribution of immune cells and cytokines to radiation-induced injury: the key roles and potential targets. Front Immunol. (2026) 17:1785418. 10.3389/fimmu.2026.1785418 42131350 PMC13160770

[7] Jaiswal J. Mesenchymal stem cell extracellular vesicles ameliorate radiation-caused dry mouth via modulating immune balance and cell metabolism. Stem Cell Res Ther. (2025) 16:579. 10.1186/s13287-025-04709-4 41272785 PMC12636160

[8] Linard C Ropenga A Vozenin-Brotons MC Chapel A Mathe D. Abdominal irradiation increases inflammatory cytokine expression and activates NF-Kappab in rat Ileal Muscularis layer. Am J Physiol Gastrointest Liver Physiol. (2003) 285:G556–565. 10.1152/ajpgi.00094.2003 12909564

[9] Buss LG Rheinheimer BA Limesand KH. Radiation-induced changes in energy metabolism result in mitochondrial dysfunction in salivary glands. Sci Rep. (2024) 14:845. 10.1038/s41598-023-50877-9 38191641 PMC10774336

[10] Muller K Meineke V. Radiation-induced alterations in cytokine production by skin cells. Exp Hematol. (2007) 35:96–104. 10.1016/j.exphem.2007.01.017 17379094

[11] Lierova A. Cytokines and radiation-induced pulmonary injuries. J Radiat Res. (2018) 59:709–53. 10.1093/jrr/rry067 30169853 PMC6251431

[12] Sharabi AB Lim M DeWeese TL Drake CG. Radiation and checkpoint blockade immunotherapy: radiosensitisation and potential mechanisms of synergy. Lancet Oncol. (2015) 16:e498–509. 10.1016/S1470-2045(15)00007-8 26433823

[13] Diesch T Filippi C Fritschi N Filippi A Ritz N. Cytokines in saliva as biomarkers of oral and systemic oncological or infectious diseases: a systematic review. Cytokine. (2021) 143:155506. 10.1016/j.cyto.2021.155506 33846070

[14] Noureldin AAK Sones A Mendoza L Mancl L Milgrom P. Effectiveness of silver diamine fluoride in preventing radiation-induced caries: a pilot study. J Am Dent Assoc. (2026). 10.1016/j.adaj.2026.05.008 42461172

[15] Bhattarai KR Kim HR Chae HJ. Compliance with saliva collection protocol in healthy volunteers: strategies for managing risk and errors. Int J Med Sci. (2018) 15:823–31. 10.7150/ijms.25146 30008593 PMC6036086

[16] Palucka AK Coussens LM. The basis of oncoimmunology. Cell. (2016) 164:1233–47. 10.1016/j.cell.2016.01.049 26967289 PMC4788788

[17] Medzhitov R. Origin and physiological roles of inflammation. Nature. (2008) 454:428–35. 10.1038/nature07201 18650913

[18] Aggarwal BB. Signalling pathways of the TNF superfamily: a double-edged sword. Nat Rev Immunol. (2003) 3:745–56. 10.1038/nri1184 12949498

[19] Moore KW De Waal Malefyt R Coffman RL O'Garra A. Interleukin-10 and the interleukin-10 receptor. Annu Rev Immunol. (2001) 19:683–765. 10.1146/annurev.immunol.19.1.683 11244051

[20] Nakamura T Mizuno S. The discovery of hepatocyte growth factor (HGF) and its significance for cell biology, life sciences and clinical medicine. Proc Jpn Acad Ser B Phys Biol Sci. (2010) 86:588–610. 10.2183/pjab.86.588 20551596 PMC3081175

[21] Wynn TA. Type 2 cytokines: mechanisms and therapeutic strategies. Nat Rev Immunol. (2015) 15:271–82. 10.1038/nri3831 25882242

[22] Schmitz J. IL-33, an interleukin-1-like cytokine that signals via the IL-1 receptor-related protein ST2 and induces T helper type 2-associated cytokines. Immunity. (2005) 23:479–90. 10.1016/j.immuni.2005.09.015 16286016

[23] Cayrol C Girard JP. Interleukin-33 (IL-33): a nuclear cytokine from the IL-1 family. Immunol Rev. (2018) 281:154–68. 10.1111/imr.12619 29247993

[24] Lira-Junior R Akerman S Gustafsson A Klinge B Bostrom EA. Colony stimulating factor-1 in saliva in relation to age, smoking, and oral and systemic diseases. Sci Rep. (2017) 7:7280. 10.1038/s41598-017-07698-4 28779164 PMC5544729

[25] Bland JMA Altman DG. Statistics notes: the use of transformation when comparing two means. Br Med J. (1996) 312:1153. 10.1136/bmj.312.7039.1153 PMC2350653 8620137

[26] Szabo YZ Slavish DC. Measuring salivary markers of inflammation in health research: a review of methodological considerations and best practices. Psychoneuroendocrinology. (2021) 124:105069. 10.1016/j.psyneuen.2020.105069 33316694 PMC8412951

[27] Rinderknecht C. Associations between salivary cytokines and oral health, age, and sex in healthy children. Sci Rep. (2022) 12:15991. 10.1038/s41598-022-20475-2 36163488 PMC9512800

[28] Hai B Zhao Q Deveau MA Liu F. Delivery of sonic hedgehog gene repressed irradiation-induced cellular senescence in salivary glands by promoting DNA repair and reducing oxidative stress. Theranostics. (2018) 8:1159–67. 10.7150/thno.23373 29464006 PMC5817117

[29] Zhao Q. Transient activation of the hedgehog-gli pathway rescues radiotherapy-induced dry mouth via recovering salivary gland resident macrophages. Cancer Res. (2020) 80:5531–42. 10.1158/0008-5472.CAN-20-0503 32998998 PMC7744395

[30] Park H Kim CH Jeong JH Park M Kim KS. GDF15 contributes to radiation-induced senescence through the ROS-mediated p16 pathway in human endothelial cells. Oncotarget. (2016) 7:9634–44. 10.18632/oncotarget.7457 26909594 PMC4891072

[31] Liu J. GDF15 participates in epithelial cell senescence in radiation-induced lung injury through the ERK1/2-p16 signaling pathway. PLoS One. (2026) 21:e0350042. 10.1371/journal.pone.0350042 42263110 PMC13249152

[32] Yoon YJ Shin HS Lim JY. A hepatocyte growth factor/MET-induced antiapoptotic pathway protects against radiation-induced salivary gland dysfunction. Radiother Oncol. (2019) 138:9–16. 10.1016/j.radonc.2019.05.012 31136962

[33] Soto-Gamez A. Mesenchymal stem cell-derived HGF attenuates radiation-induced senescence in salivary glands via compensatory proliferation. Radiother Oncol. (2024) 190:109984. 10.1016/j.radonc.2023.109984 37926332

[34] Gupta N. Radiation-induced dental caries, prevention and treatment—a systematic review. Natl J Maxillofac Surg. (2015) 6:160–6. 10.4103/0975-5950.183870 27390489 PMC4922225

[35] Shah SD. The role of salivary diagnostics in early detection of systemic and oral diseases: a comprehensive review. Cureus. (2025) 17:e100313. 10.7759/cureus.100313 41613689 PMC12848634

[36] Huang L Luo F Deng M Zhang J. The relationship between salivary cytokines and oral cancer and their diagnostic capability for oral cancer: a systematic review and network meta-analysis. BMC Oral Health. (2024) 24:1044. 10.1186/s12903-024-04840-3 39237889 PMC11378403

[37] Russo N. Cytokines in saliva increase in head and neck cancer patients after treatment. Oral Surg Oral Med Oral Pathol Oral Radiol. (2016) 122:483–90 e481. 10.1016/j.oooo.2016.05.020 27554375 PMC5031552

[38] Bossi P. Salivary cytokine levels and oral mucositis in head and neck cancer patients treated with chemotherapy and radiation therapy. Int J Radiat Oncol Biol Phys. (2016) 96:959–66. 10.1016/j.ijrobp.2016.08.047 27745982

[39] Principe S Dikova V Bagan J. Salivary cytokines in patients with head and neck cancer (HNC) treated with radiotherapy. J Clin Exp Dent. (2019) 11:e1072–7. 10.4317/jced.56318 31700580 PMC6825732

[40] Hu L. Transient activation of hedgehog signaling inhibits cellular senescence and inflammation in radiated swine salivary glands through preserving resident macrophages. Int J Mol Sci. (2021) 22:13493. 10.3390/ijms222413493 34948290 PMC8708934

[41] McKendrick JG. CSF1R-dependent macrophages in the salivary gland are essential for epithelial regeneration after radiation-induced injury. Sci Immunol. (2023) 8:eadd4374. 10.1126/sciimmunol.add4374 37922341 PMC7619197

