# Supplementary material for: Changes of salivary cytokines in head and neck cancer patients following radiotherapy
Source: Front Oral Health. 2026 Oct 14;7:1969672. doi: 10.3389/froh.2026.1969672 (PMC13713834; doi:10.3389/froh.2026.1969672)
Supplement: Supplementary file 1 [file Datasheet1.pdf]

Supplementary data

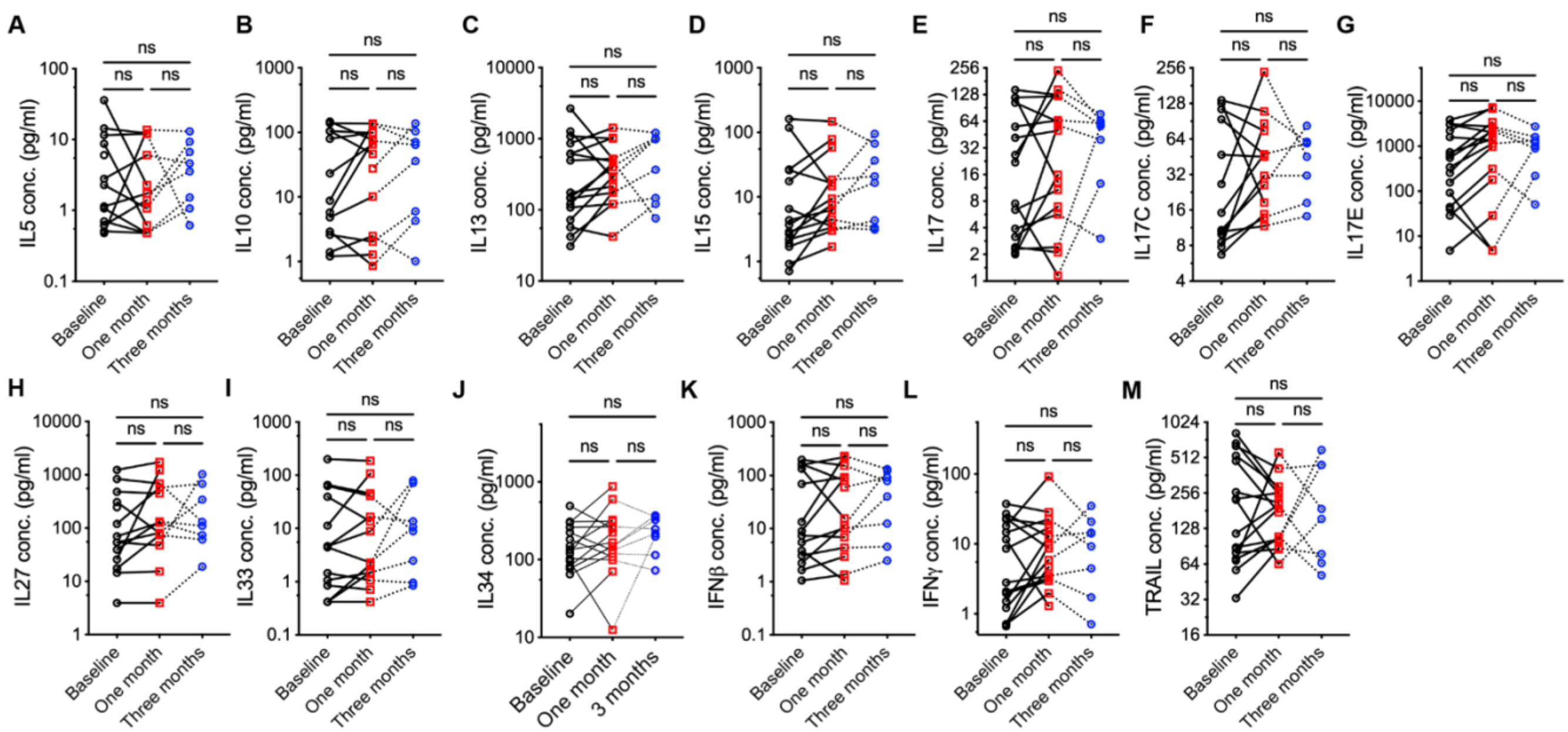

Supplementary Figure S1. Salivary levels of cytokines without significant changes after radiotherapy.

As in Figure 2, geometric means were compared between each time points using a one way mixed-effects model. ns: not significant.

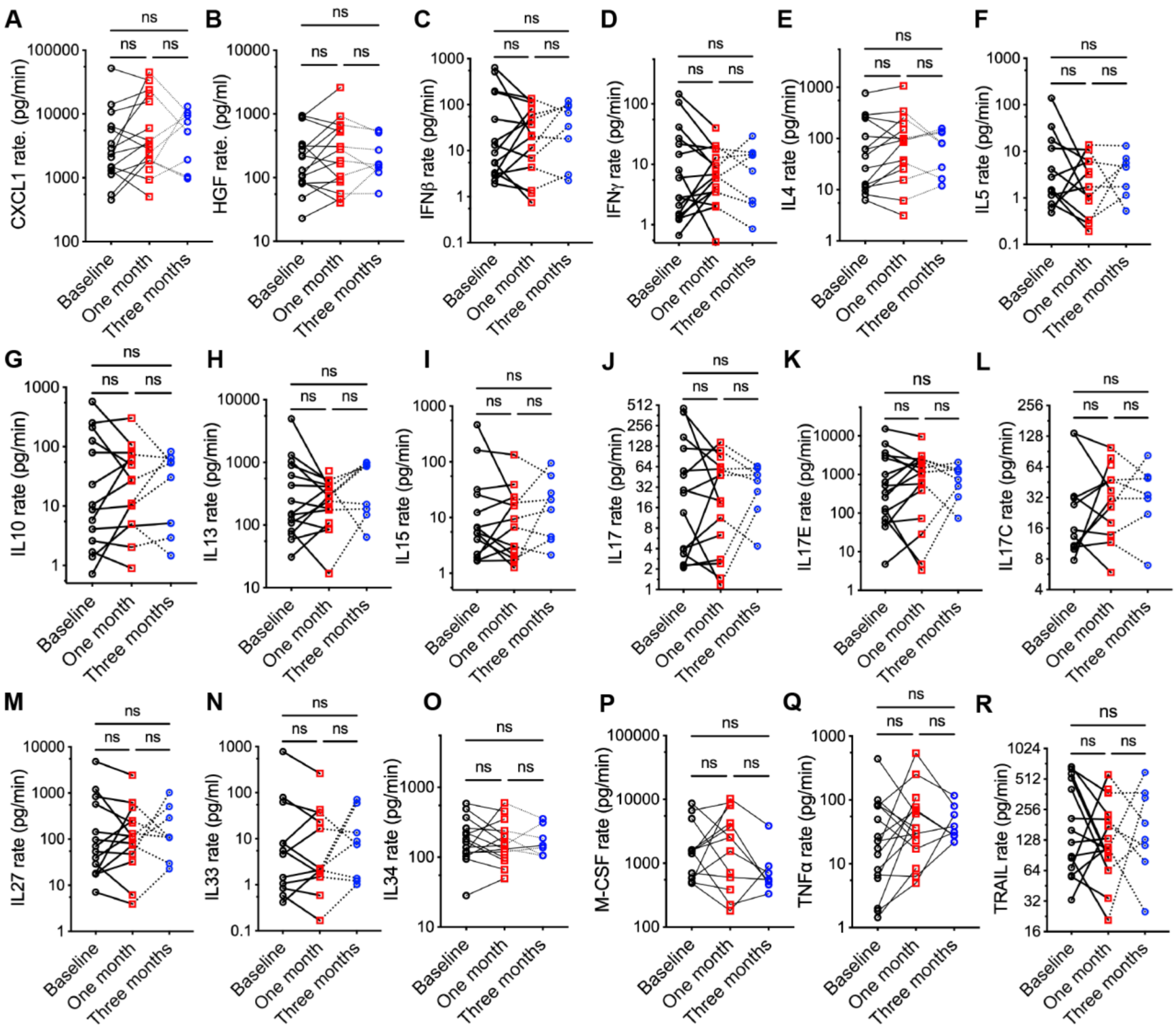

Supplementary Figure S2. Rate-adjusted salivary levels of cytokines without significant changes after radiotherapy.

As in Figure 3, geometric means were compared between each time points using a one way mixed-effects model. ns: not significant.
